# Real-life analysis on safety and efficacy of asciminib for ponatinib pretreated patients with chronic myeloid leukemia

**DOI:** 10.1007/s00277-022-04932-6

**Published:** 2022-08-23

**Authors:** A. Luna, L. Pérez-Lamas, C. Boque, P. Giraldo, B. Xicoy, C. Ruiz Nuño, M. Moreno Vega, A. Alvarez-Larrán, A. Salamanca, A. García-Noblejas, F. Vall-Llovera, L. Villalon, N. De las Heras, E. Ramila, M. Pérez-Encinas, B. Cuevas, R. Perez-Lopez, F. Sanchez-Guijo, A. Jiménez-Velasco, S. Lakhwani, L. Felipe Casado, A. Rosell, A. Escola, M. J. Fernández, C. Garcia-Hernandez, C. Cervero, E. Mora, M. Sagüés, S. Suarez-Varela, P. Vélez, P. Carrascosa Mastell, R. F. Bitaube, L. Serrano, M. Cortes, J.A Vera Goñi, J. L. Steegmann, V. Gomez Garcia de Soria, J. M. Alonso-Dominguez, M. Colorado Araujo, A. Paz Coll, J.C Hernandez-Boluda, V. García-Gutiérrez

**Affiliations:** 1 Hematology Department, Hospital Universitario Ramón y Cajal, IRYCIS, Universidad de Alcala, Km 9,1. 28034, Madrid, Spain; 2 Institut Catala d’Oncologia, Hospital Duran Y Reynals, L’Hospitalet de Llobregat, Barcelona, Spain; 3 Hospital Quirón Zaragoza, Zaragossa, Spain; 4 Institut Català d’Oncologia-Hospital Germans Trias I Pujol, Badalona, Spain; 5 Hospital Regional Universitario de Málaga, Málaga, Spain; 6 Hospital Doctor José Molina Orosa de Lanzarote, Arrecife, Spain; 7 Hospital Clinic de Barcelona, Barcelona, Spain; 8 Hospital de Jerez de La Frontera, Jerez de la Frontera, Spain; 9 Hospital Universitario de La Princesa, Madrid, Spain; 10 Hospital Mutua de Terrassa, Terrassa, Spain; 11 Hospital Universitario Fundación Alcorcón, Alcorcón, Spain; 12 Hospital Universitario de León, León, Spain; 13 Hospital Parc Tauli, Sabadell, Spain; 14 Hospital Clínico Universitario de Santiago de Compostela, Santiago de Compostela, Spain; 15 Hospital Universitario de Burgos, Burgos, Spain; 16 Hospital Universitario Clínico Virgen de La Arrixaca, El Palmar, Spain; 17 Department of Medicine, University of Salamanca, Hematology Department, IBSAL-University Hospital of Salamanca, Salamanca, Spain; 18 Hospital Universitario Carlos Haya, Málaga, Spain; 19 Hospital Universitario de Canarias, La Laguna, Spain; 20 Hospital Virgen de La Salud, Toledo, Spain; 21 Hospital Virgen de La Victoria, Málaga, Spain; 22 Hospital Provincial de Castellón, Castellon, Spain; 23 Hospital Doctor Peset, Valencia, Spain; 24 Hospital General de Alicante, Alicante, Spain; 25 Hospital Virgen de La Luz, Cuenca, Spain; 26 Hospital Universitario Y Politécnico La Fe, Valencia, Spain; 27 Hospital General de Castellón, Castellón De La Plana, Spain; 28 Hospital de Granollers, Granollers, Spain; 29 Hospital Virgen Macarena, Seville, Spain; 30 Hospital Universitario Fundación Jiménez Díaz, Madrid, Spain; 31 Hospital Universitario Marqués de Valdecilla, Santander, Spain; 32 Hospital Universitario Puerto Real, Puerto Real, Spain; 33 Hospital Clínico Universitario-INCLIVA, Valencia, Spain; 34 University of Valencia, Valencia, Spain

**Keywords:** Leukemia, Inhibitors, Asciminib

## Abstract

Failure of second-generation tyrosine kinase inhibitors (2GTKI) is a challenging situation in patients with chronic myeloid leukemia (CML). Asciminib, recently approved by the US Federal Drug Administration, has demonstrated in clinical trials a good efficacy and safety profile after failure of 2GTKI. However, no study has specifically addressed response rates to asciminib in ponatinib pretreated patients (PPT). Here, we present data on responses to asciminib from 52 patients in clinical practice, 20 of them (38%) with prior ponatinib exposure. We analyzed retrospectively responses and toxicities under asciminib and compared results between PPT and non-PPT patients.

After a median follow-up of 30 months, 34 patients (65%) switched to asciminib due to intolerance and 18 (35%) due to resistance to prior TKIs. Forty-six patients (88%) had received at least 3 prior TKIs. Regarding responses, complete cytogenetic response was achieved or maintained in 74% and 53% for non-PPT and PPT patients, respectively. Deeper responses such as major molecular response and molecular response 4.5 were achieved in 65% and 19% in non-PPT versus 32% and 11% in PPT, respectively. Two patients (4%) harbored the T315I mutation, both PPT.

In terms of toxicities, non-PPT displayed 22% grade 3–4 TEAE versus 20% in PPT. Four patients (20% of PPT) suffered from cross-intolerance with asciminib as they did under ponatinib.

Our data supports asciminib as a promising alternative in resistant and intolerant non-PPT patients, as well as in intolerant PPT patients; the resistant PPT subset remains as a challenging group in need of further therapeutic options.

## Introduction

In recent years, chronic myeloid leukemia (CML) marked a major milestone in cancer targeted therapies after the development of tyrosine-kinase inhibitors (TKIs)[1], with imatinib as a pioneer, leveling prognosis in CML to a normal life expectancy[2].

The further development of new-generation TKIs set a new era for precision medicine: initially second-generation TKI (2GTKI) such as dasatinib[3], nilotinib[4], bosutinib[5], and thereafter ponatinib as a third-generation TKI[6]; tailored treatment in CML became reality[7], highly individualized by disease history, adverse effects under previous TKIs, and underlying mutations.

Unfortunately, an important proportion of patients fail all currently available TKIs, due to resistance or intolerance: approximately 50% of patients with first line imatinib will develop intolerance or resistance, whereas for 2GTKI, 30–40% will need a change of therapy[8]. In this scenario, asciminib has been developed as a first-in-class BCR::ABL1 inhibitor which Specifically Targets the ABL Myristoyl Pocket (STAMP inhibitor), thus having a different mechanism of action to the previous TKIs. Asciminib binds specifically to a myristoyl pocket in the ABL kinase, which is a distinct site from the ATP binding area of the kinase, thereby restoring an autoregulatory mechanism lost in BCR::ABL1 and stabilizing an assembled inactive state of the protein[9]. For this reason, asciminib has the potential to overcome resistance to prior TKIs, including the T315 mutation, and with the possibility of dual inhibition of BCR::ABL1 in combination with ATP-binding TKIs[10]. Unlike other non-selective TKIs, asciminib is specific for ABL kinases, and this should translate into an improved tolerability[11]. Asciminib has been promisingly evaluated in a phase I study in patients with Ph-positive leukemia failing prior TKIs, showing clinical efficacy in CML patients with previous exposure to > 2 TKIs, including patients harboring the drug-resistant T315I mutation[12]. Furthermore, asciminib showed superiority compared to bosutinib in a phase III trial with CML patients resistant to at least 2 TKIs[13]. Of interest, previous exposure to ponatinib was not mandatory in the mentioned clinical trials.

Although asciminib has recently been approved in the USA, there is still a lack of information regarding its use in the real-world setting, including the benefit of asciminib in patients previously exposed to ponatinib. Accessibility of this information would be key in the context of the release of the drug, helping clinicians and hospitals tailor this new therapeutic option with accuracy for the patients in need.

Our aim is to discern differences in safety and efficacy in patients under asciminib with and without prior ponatinib treatment in real-world practice setting.

## Methods

We gathered data from 52 CML patients treated with asciminib between October 2018 and July 2021 in 33 Spanish institutions joined to the Spanish CML group (GELMC). Asciminib was provided by a managed-access program (MAP) allowed by Novartis. MAP requests were independently reviewed by licensed treating physicians to confirm that the following criteria were met: treatment need of a serious or life-threatening disease lacking commercially available options, patients had to be ineligible or unable to participate in a clinical trial, and the request should be in alignment with all applicable local laws and regulations. The study was approved by the Spanish Drug Agency and the Ethics Committee of the Hospital Universitario Ramón y Cajal (Madrid, Spain), with informed consent being obtained from all patients. BCR::ABL1 analysis was not centralized, but all samples were analyzed in EUTOS accredited laboratories. Mutational status tests were performed in all patients after switching to a second line of TKI; no mutational status studies were performed during or after asciminib treatment.

Response analysis was performed following the European Leukemia Net 2020 recommendations[14]. Treatment failure to prior TKIs was defined either as resistance (BCR::ABL1 increase despite optimal TKI dosage) or intolerance (unacceptable toxicity leading to TKI termination). Variables studied to identify factors associated with response to asciminib included resistance versus intolerance, previous use of ponatinib, prior complete cytogenetic response (CCyR) status, and presence of BCR::ABL1 mutations. Cumulative response was defined as reaching or at least maintaining previous response. Event-free survival (EFS) was defined as time from first dose of asciminib to on-treatment death, progression to advanced phase, confirmed loss of CCyR, loss of complete hematologic response (CHR), treatment discontinuation for any reason (intolerance or lack of efficacy), or death for any reason. Treatment emergent adverse events (TEAEs) were graded according to the National Cancer Institute Common Terminology Criteria for Adverse Events Version 5.0. Study data were collected and managed using REDCap electronic data capture tools hosted at Ramón y Cajal Hospital. Data analysis was performed with SPSS Version 27.0.

## Results

The baseline characteristics of the study series are displayed in Table 1. Twenty out of the 52 patients (38%) had been previously treated with ponatinib, hereinafter referred to as PPT. The median time of asciminib treatment was 11.7 months for the entire cohort (range 2–30 months): median time on previous TKIs treatment until the start of asciminib was 121 months. Patients were heavily pretreated, with 46 (88%) having received 3 or more TKIs before asciminib. A total of 13 patients (25%) had baseline BCR::ABL1 mutations, and 2 of them harbored the T315I mutation. Previous time on TKIs before switch to asciminib was 30 months. Asciminib was prescribed due to intolerance to prior TKIs in 34 patients (65%) and due to resistance in the remaining 18 (35%). The starting asciminib dose was 40 mg BID except in the two cases with the T315I mutation, who received 200 mg BID.

**Table 1:** Patients’ characteristics considering prior ponatinib use

	Non-ponatinib pretreated patients (*n* = 32)	Ponatinib pretreated patients (*n* = 20)
Median age at data collection, yr (range)	69 (37–91)	57 (43–85)
Median age at diagnosis, yr (range)	56 (27–87)	48 (20–70)
Female sex, ***n*** (%)	17 (53)	12 (60)
Median time on previous TKIs, months (range)	89 (6–305)	91 (10–256)
Disease stage before asciminib, ***n*** (%)		
Chronic phase	32 (100)	19 (95)
Accelerated phase	0	1 (5)
Blast phase	0	0
Sokal risk, ***n*** (%)		
Low	15 (47)	9 (45)
Intermediate	8 (25)	4 (20)
High	5 (16)	4 (20)
Unknown	4 (13)	3 (15)
Switch to asciminib due to intolerance, ***n*** (%)	24 (75)	10 (50)
Switch to asciminib due to resistance, ***n*** (%)	8 (25)	10 (50)
TKI at diagnosis, ***n*** (%)		
Imatinib	26 (81)	15 (75)
Dasatinib	1 (3)	3 (15)
Nilotinib	5 (16)	1 (5)
Bosutinib	0	1 (5)
≥ 3 prior TKI lines, ***n*** (%)	28 (88)	12 (60)
BCR::ABL1 mutations, ***n*** (%)	6 (19)	7 (35)
T315I ***n*** (%)	0	2 (10)

### Efficacy

Switching to asciminib was mainly due to intolerance in the non-PPT group (75% of cases), while in the PPT group it was balanced as half intolerant and half resistant.

In the whole series (PPT and non-PPT), cumulative response rates of complete hematological (CHR), complete cytogenetic (CCyR), and major molecular response (MMR) were 92%, 66%, and 52%, respectively.

After excluding patients in CCyR or MMR at baseline, the cumulative rates of CCyR and MMR were 42% (11/26) and 40% (15/38), respectively. In resistant and intolerant patients, probabilities to obtain CCyR were 41% (7/17) versus 35% (6/17) and for MMR: 79% (26/33) versus 61% (20/33), respectively.

Considering prior exposure to ponatinib (Table 2), rates of achieving or maintaining response in the non-PPT group were, for CCyR, MMR, and molecular response grade 4.5 (MR 4.5): 74%, 65%, and 19%, respectively; and 52%, 32%, and 11%, respectively, in the PPT group.

**Table 2:** Responses to asciminib regarding baseline response and prior use of ponatinib

	Resistant (*n* = 17)		Intolerant (*n* = 33)		Total (*n* = 50*)		
	Non PPT (*n* = 8)	PPT (*n* = 9)	Non PPT (*n* = 23)	PPT (*n* = 10)	Non PPT (*n* = 31)	PPT (*n* = 19)	
CHR^a^	8/8 (100%)	5/9 (55.5%)	23/23 (100%)	10/10 (100%)	31/31 (100%)	15/19 (79%)	46/50 (92%)
CCyR^a^	3/8 (37.5%)	4/9 (44.4%)	20/23 (87%)	6/10 (60%)	23/31 (74.2%)	10/19 (52.6%)	33/50 (66%)
MMR^a^	3/8 (37.5%)	3/9 (33.3%)	17/23 (74%)	3/10 (30%)	20/31 (64.5%)	6/19 (31.6%)	26/50 (52%)
MR4.5^a^	0/8 (0%)	1/9 (11.1%)	6/23 (26%)	1/10 (10%)	6/31 (19.4%)	2/19 (10.5%)	8/50 (16%)
Patients without response at baseline							
CCyR^b^	2/7 (28.6%)	2/7 (28.6%)	6/8 (75%)	1/4 (25%)	8/15 (53.3%)	3/11 (27.3%)	11/26 (42.3%)
MMR^b^	2/7 (28.6%)	2/8 (25%)	10/16 (62.5%)	1/7 (14.3%)	12/23 (52.2%)	3/15 (20%)	15/38 (39.5%)
MR4.5^b^	0/8 (0%)	1/9 (11.1%)	6/23 (26%)	1/10 (10%)	6/31 (19.4%)	2/19 (10.5%)	8/50 (16%)

*PPT prior ponatinib treatment, CHR complete hematological response, CCyR complete cytogenetic response, MMR major molecular response, MR4.5 detectable disease with BCR::ABL1IS* < *0.0032%*

^*a*^*Patients with CHR, CCyR, MMR, or MR4.5 at baseline were evaluable for hematologic, cytogenetic, or molecular response and were considered responders if they maintained their response*

^*b*^*Evaluable patients without a CCyR, MMR, or MR4.5 at baseline*

^*^Two patients of the 52 cohort were excluded for the response analysis due to short follow-up and missing data on response

Moreover, probability of improving baseline responses was 53%, 57%, and 19% towards CCyR, MMR, and MR4.5 for the non-PPT versus the PPT group reaching 27%, 20%, and 11%, respectively.

Comparing probabilities for maintaining or improving responses regarding prior resistant or intolerant status: in the non-PPT group, intolerant patients displayed: 87% CCyR, 74% MMR, and 26% MR4.5, whereas resistant non-PPT showed 38% CCyR, 38% MMR, and 0% MR4.5, respectively. In the PPT group, prior intolerant patients displayed: 60% CCyR, 30% MMR, and 10% MR4.5, whereas resistant patients showed: 44% CCyR, 33% MMR, and 11% MR4.5, respectively.

Considering patients with lack of response, the resistant group displayed the worst outcomes, regardless of prior ponatinib use. Ponatinib use per se does not seem to have an impact on response in our group, unlike the unresponsiveness of the whole resistant group.

Concerning baseline mutational status (Table 3), 6 patients in the non-PPT group (19%) and 7 patients in the PPT group (35%) had BCR::ABL1 mutations. The mutational pattern was very heterogeneous between patients, displaying mutations such as: E255K, E255V, exon 7 deletion, F311L, F359V, G250E, G459G, I313T, M244V, T257C, V299L, and Y253H. Two patients displayed exon 7 deletions, considered a potential artifact in the genetic analysis. Their individual responses are displayed in Table 3. In the PPT group, 6 out of 8 (75%) patients with mutations maintained or improved responses, in line with the general group. In these PPT-mutated, 4 patients (50%) came from the intolerant group and 4 (50%) from the resistance group.

**Table 3:** Best responses with asciminib in patients with BCR::ABL1 mutations, considering prior use of ponatinib

BCR::ABL1-mutated patients	Best response reached after asciminib
Ponatinib pretreated patients	
Patient 1: T315I	CHR
Patient 2: T315I	MR^4.5^
Patient 3: Y253H, E255V, V299L, F317L	CHR
Patient 4: E255K	CHR
Patient 5: V304A, A362S	CHR
Patient 6: T257C	CHR
Patient 7: I313T	CCyR
Non-ponatinib pretreated patients	
Patient 8: M244V	PCyR
Patient 9: F311L, E459G	PCyR
Patient 10: F359V, exon 7 deletion	MMR
Patient 11: F311L	MMR
Patient 12: E255V, G250E	MR^4^
Patient 13: Exon 7 deletion	MR^4^

Two patients (4%) harbored the T315I mutation, both in the PPT-resistant group. One patient improved the depth of response from MR4 to MR4.5, whereas the other lost CHR and discontinued asciminib.

### Safety

**Fig. 1:**
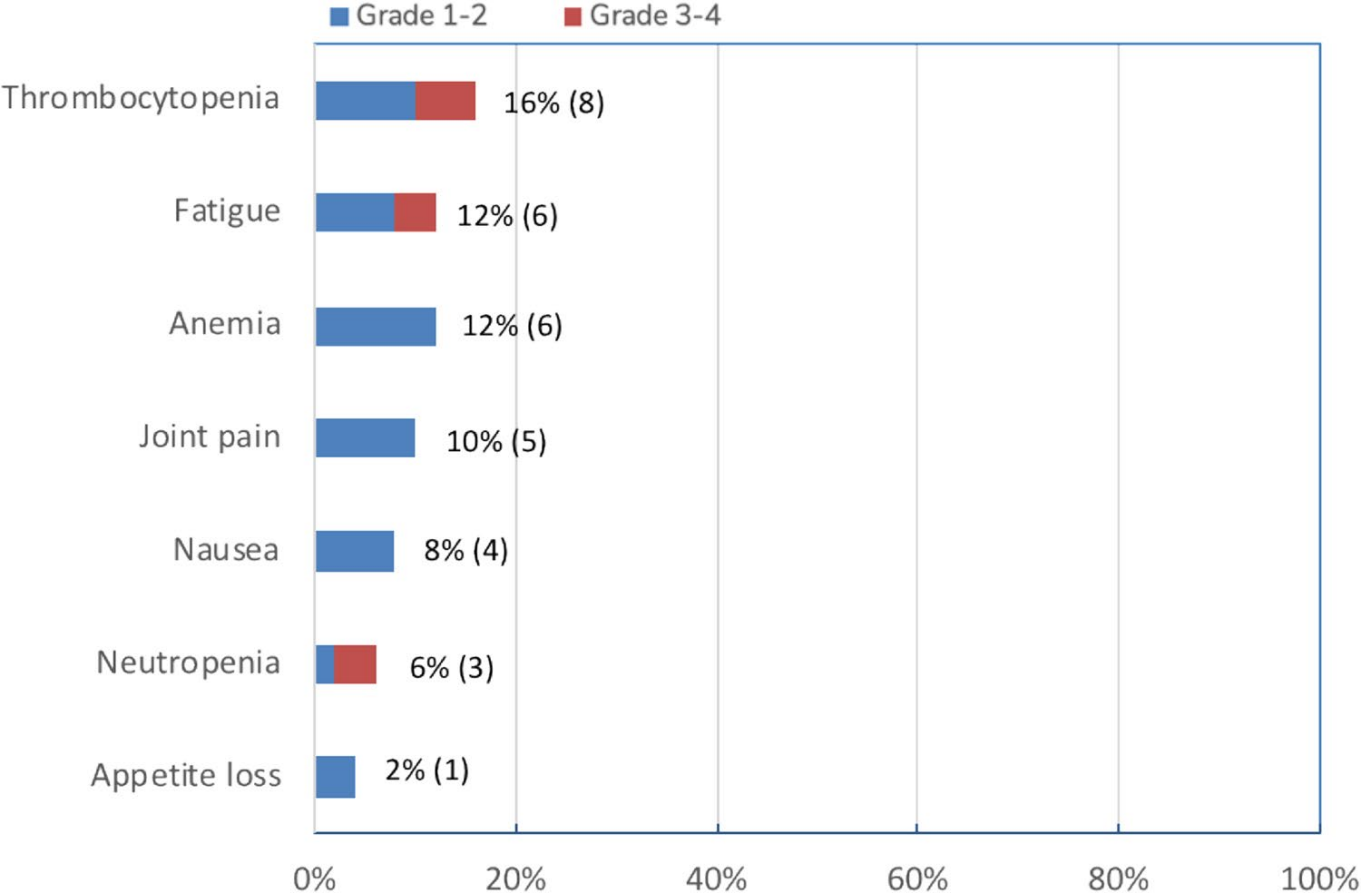
Adverse events in the entire asciminib cohort, stacked according to severity

Overall, half of the patients suffered from any TEAE, while 19% (10 patients) had grade 3–4 TEAE, with thrombocytopenia being the most common (Fig. 1). No cardiovascular events were reported. Considering subgroups, non-PPT displayed 22% grade 3–4 TEAE versus 20% in PPT. Prior ponatinib and intolerance did not play a strong role: 12/24 (50%) displayed side effects in the non-PPT-intolerant subgroup, 4 of them grade 3–4 (33%), whereas in the PPT-intolerant subgroup 5/10 patients (50%) suffered from toxicities, 2 of them grade 3–4: (20%), grade 4 thrombocytopenia and grade 3 pancreatitis, accordingly.

Regarding cross-intolerance in the PPT group, only 4 patients (20%) presented the same type of TEAE with asciminib: grade 4 thrombocytopenia, grade 3 pancreatitis, grade 1 anemia, and grade 1 lumbar pain.

**Fig. 2:**
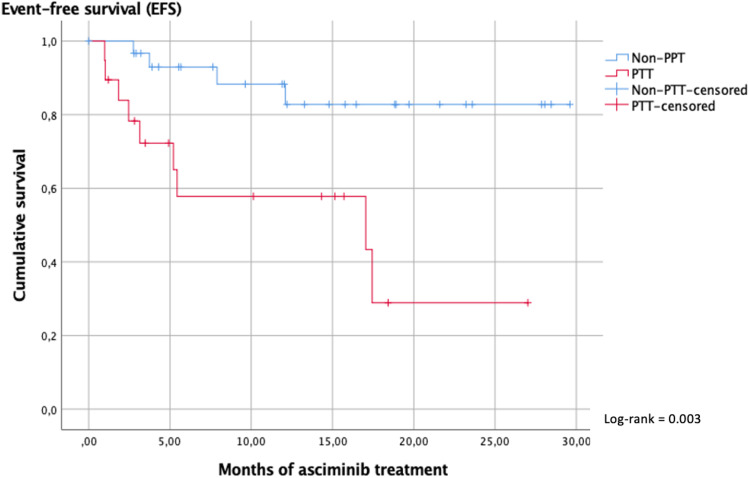
Event-free survival considering prior ponatinib treatment. Event was defined as: on-treatment death, progression to advanced phase, confirmed loss of complete cytogenetic response (CCyR), loss of complete hematologic response (CHR), treatment discontinuation for any reason (intolerance or lack of efficacy), or death for any reason

Discontinuation rate of asciminib was higher in PPT patients (45% in PPT versus 13% in non-PPT). As shown in Fig. 2, the median EFS was 17 months in PTT and was not reached in non-PPT patients, with a plateau of the survival curve at 82%.

**Fig. 3:**
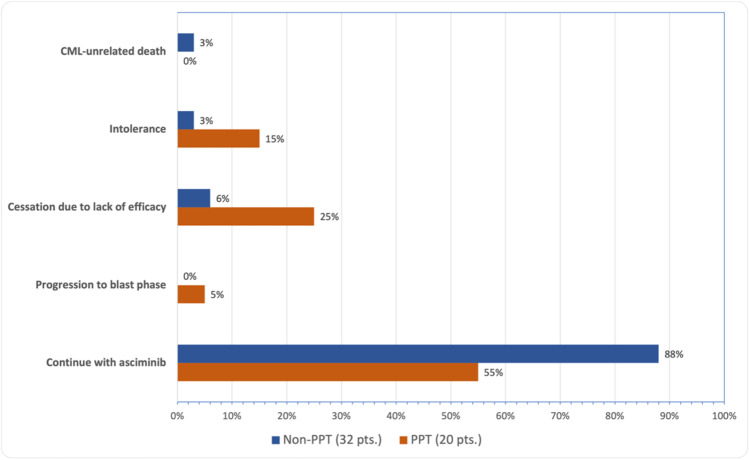
Reasons for asciminib discontinuation at the end of follow-up, considering prior ponatinib use

After a total follow-up period of 30 months (Fig. 3), 39 patients (75%) continued receiving asciminib, while the remaining discontinued treatment due to: intolerance (4 patients), loss of efficacy (7 patients), progression to blast crisis (1 patient), and CML-unrelated death (1 patient). About those progressing despite asciminib (8 patients, 1 to blast phase): 3/8 patients (38%) harbored kinase mutations: patient 1: T315I, patient 4: E255K, and patient 5: V304A + A362S.

## Discussion

Ponatinib is a very potent TKI capable of overcoming resistance to multiple BCR::ABL1 mutations, such as T315I. Consequently, ponatinib-resistant patients have very limited treatment alternatives[15]. Asciminib has been recently approved in the USA for patients failing to at least 2 TKIs after showing benefits in the ASCEMBL trial, compared to bosutinib in that situation. Although ponatinib is considered as the preferred treatment option for CML resistance patients, asciminib has not been directly compared to ponatinib[8]. We believe that the data presented here could help physicians to make treatment decisions once asciminib becomes broadly approved.

In a previous report, we showed promising preliminary results in a cohort of 31 patients treated with asciminib in the real-life setting[16]. In the current study, we have expanded the number of patients and the observation period of the series, aiming to compare the results of asciminib according to prior ponatinib exposure.

Overall, our data demonstrates a higher efficacy of asciminib in non-PPT patients, who displayed globally better outcomes than PPT: CCyR 74% versus 53% and MMR 66% versus 32%, respectively. Importantly, these differences impacted in EFS. However, after analyzing the responses rates in different cohorts, the group that benefited the most from asciminib was the non-PPT intolerant. Of interest, as it might be expected, a higher response rate was not observed in resistant non-PPT patients compared to the resistant PPT group. These data, due to the low number of patients included in each group, should be taken with caution, warranting to be further researched in subsequent studies. Considering mutational status, PPT-resistant patients did not display BCR::ABL1 mutations more often than other subgroups, apart from the T315I mutation. This can be due to the heterogeneity of the mutational spectrum in our sample and may hint that some resistance mechanisms are not exclusively explained by BCR::ABL1 mutations.

Our response rates are comparable to those of the prior phase I clinical trial including 150 CML patients switching to asciminib due to resistance or intolerance to other TKIs, with 24 patients having been exposed to ponatinib[12]. Overall, we obtained similar outcomes comparing to those in the trial for achieving or maintaining CCyR (66% in ours versus 70% in the trial) and MMR (52% in ours versus 48% in the trial); however, in the PPT group, our rate of patients who achieved a MMR is much lower than that described in the clinical trial, with only 3/15 achieving MMR (20%) in our series versus 8/14 (57%) in the trial for the non T315I mutation group. This could also be explained by the fact that the PPT cohort had more resistant patients (50%) than the non-PPT cohort (25%). In this phase I study, authors did not describe the number of patients with resistance to last TKI in the PPT group. Nevertheless, treatment responses in the global population were clearly influenced by previous response to last TKI, with 30 of 40 patients (75%) with a baseline BCR::ABL1 IS < 1% achieving MMR by 12 months versus 14 of 51 patients (27%) with a BCR::ABL1 IS ≥ 1%. In the ASCEMBL trial, only 41/233 (17%) had previously received ponatinib, and responses were not analyzed according to previous ponatinib exposure.

The T315I mutation has a significant impact on CML prognosis. With ponatinib as the only available drug up to now effective against this mutation, asciminib and its allosteric inhibition mechanism may prove promising. Data from the phase I trial with asciminib showed how only 1/7 patients harboring the T315I mutation with previous exposure to ponatinib obtained MMR. Thus far, in our sample the two patients with T315 mutation showed disparity, one deepening response whereas the other lost it despite receiving full dosing. Though very limited in numbers, this data may indicate that T315I remains as a negative risk factor with difficulties to be taken under control. Future directions to tackle this issue may include combinations of ponatinib and asciminib to maximize efficacy in these patients.

Regarding toxicities, we found lower incidences of TEAEs compared to mentioned clinical trials. These differences are probably related to the retrospective design of our study. In contrast, treatment discontinuation related to AEs is similar in the phase I and III trials.

Cross-intolerance happened in 4 PPT patients, as previously described. Favorably, no cardiovascular events happened in the PPT group, nor in those who had discontinued ponatinib due to such side effects. While cross-intolerance rarely occurs, likely to its different mechanism of action and higher kinase selectivity, it is important to highlight the relative short follow-up period in CML patients exposed to asciminib.

Main limitations of the study are the relative low number of patients and the retrospective design; nevertheless, ponatinib pretreated patients are well represented in our sample, with larger proportions than those in the phase I trial. Close monitoring with longer follow-up is mandatory to better establish the safety profile of the drug.

Asciminib is now available for CML patients previously treated with two or more TKIs after the results of the phase III ASCEMBL trial[13]. While asciminib showed superiority in this situation against bosutinib, no direct comparison has been made with ponatinib. Our data supports, from a real-life perspective, the previous results of asciminib in clinical trials[17]. In our series, the major benefit of asciminib seems to be the excellent safety profile, with very few patients discontinuing treatment due to side effects. This makes asciminib an excellent treatment option for those patients abandoning previous TKIs due to intolerance. However, the issue of replacing ponatinib as the most efficacious option for CML patients is still far from solved, since the ones that benefit less from asciminib seem to be those with prior ponatinib, both resistant or intolerant, or those resistant to any TKI, remaining as a challenging group in need of further therapeutic alternatives.

## References

[1] Goldman JM (2010). Chronic myeloid leukemia: a historical perspective. Semin Hematol.

[2] Bower H, Björkholm M, Dickman P, Höglund M, Lambert P, Andersson T (2016). Life expectancy of patients with chronic myeloid leukemia approaches the life expectancy of the general population. Journal of Clinical Oncology: official journal of the American Society of Clinical Oncology.

[3] Kantarjian H, Shah NP, Hochhaus A (2010). Dasatinib versus imatinib in newly diagnosed chronic-phase chronic myeloid leukemia. N Engl J Med.

[4] Saglio G, Kim DW, Issaragrisil S, le Coutre P, Etienne G, Lobo C, Pasquini R, Clark RE, Hochhaus A, Hughes TP, Gallagher N, Hoenekopp A, Dong M, Haque A, Larson RA, Kantarjian HM; ENESTnd Investigators (2010) Nilotinib versus imatinib for newly diagnosed chronic myeloid leukemia. N Engl J Med. 362(24):2251-9 10.1056/NEJMoa0912614 20525993

[5] Cortes JE, Gambacorti-Passerini C, Deininger MW, Mauro MJ, Chuah C, Kim DW, Dyagil I, Glushko N, Milojkovic D, le Coutre P, Garcia-Gutierrez V, Reilly L, Jeynes-Ellis A, Leip E, Bardy-Bouxin N, Hochhaus A, Brümmendorf TH (2018). Bosutinib versus imatinib for newly diagnosed chronic myeloid leukemia: results from the randomized BFORE trial. J Clin Oncol.

[6] Cortes JE, Kim DW, Pinilla-Ibarz J, le Coutre PD, Paquette R, Chuah C, Nicolini FE, Apperley JF, Khoury HJ, Talpaz M, DeAngelo DJ, Abruzzese E, Rea D, Baccarani M, Müller MC, Gambacorti-Passerini C, Lustgarten S, Rivera VM, Haluska FG, Guilhot F, Deininger MW, Hochhaus A, Hughes TP, Shah NP, Kantarjian HM (2018). Ponatinib efficacy and safety in Philadelphia chromosome-positive leukemia: final 5-year results of the phase 2 PACE trial. Blood.

[7] García-Gutiérrez V, Hernández-Boluda JC (2019). Tyrosine kinase inhibitors available for chronic myeloid leukemia: efficacy and safety. Front Oncol.

[8] Cortes J, Lang F (2021). Third-line therapy for chronic myeloid leukemia: current status and future directions. J Hematol Oncol.

[9] Manley PW, Barys L, Cowan-Jacob SW (2020). The specificity of asciminib, a potential treatment for chronic myeloid leukemia, as a myristate-pocket binding ABL inhibitor and analysis of its interactions with mutant forms of BCR::ABL1 kinase. Leuk Res.

[10] Wylie AA (2017). The allosteric inhibitor ABL001 enables dual targeting of BCR::ABL1. Nature.

[11] Manley PW, Barys L, Cowan-Jacob SW (2020). The specificity of asciminib, a potential treatment for chronic myeloid leukemia, as a myristate-pocket binding ABL inhibitor and analysis of its interactions with mutant forms of BCR::ABL1 kinase. Leuk Res.

[12] Hughes TP (2019). Asciminib in chronic myeloid leukemia after ABL kinase inhibitor failure. N Engl J Med.

[13] Delphine Rea, Michael J Mauro, Carla Boquimpani, Yosuke Minami, Elza Lomaia, Sergey Voloshin, Anna Grigorievna Turkina, Dong-Wook Kim, Jane F Apperley, Andre Abdo, Laura M Fogliatto, Dennis Dong Hwan Kim, Philipp le Coutre, Susanne Saussele, Mario Annunziata, Timothy P Hughes, Naeem Chaudhri, Koji Sasaki, Lynette Chee, Valentin Garcia-Gutierrez, Jorge Cortes, Paola Aimone, Alex Allepuz, Sara Quenet, Véronique Bédoucha, Andreas Hochhaus (2021) A phase 3, open-label, randomized study of asciminib, a STAMP inhibitor, versus bosutinib in CML after ≥2 prior TKIs. Blood 10.1182/blood.2020009984 PMC9728405 34407542

[14] Hochhaus A, Baccarani M, Silver RT (2020). European LeukemiaNet 2020 recommendations for treating chronic myeloid leukemia. Leukemia.

[15] Hochhaus A, Breccia M, Saglio G, García-Gutiérrez V, Réa D, Janssen J, Apperley J (2020). Expert opinion-management of chronic myeloid leukemia after resistance to second-generation tyrosine kinase inhibitors. Leukemia.

[16] Garcia-Gutiérrez V, Luna A, Alonso-Dominguez JM (2021). Safety and efficacy of asciminib treatment in chronic myeloid leukemia patients in real-life clinical practice. Blood Cancer J.

[17] Breccia M, Colafigli G, Scalzulli E, Martelli M (2021). Asciminib: an investigational agent for the treatment of chronic myeloid leukemia. Expert Opin Investig Drugs.

